# Role of physiotherapy in the mobilization of patients with spinal cord injury undergoing human embryonic stem cells transplantation

**DOI:** 10.1186/s40169-016-0122-5

**Published:** 2016-10-20

**Authors:** Geeta Shroff, Dipin Thakur, Varun Dhingra, Deepak Singh Baroli, Deepanshu Khatri, Rahul Dev Gautam

**Affiliations:** 1 Nutech Mediworld, H-8, Green Park Extension, New Delhi, 110016 India; 2 Nutech Mediworld, New Delhi, 110016 India

**Keywords:** Tetraplegics, Paraplegics, Physical therapy, Human embryonic stem cell therapy, Orthosis

## Abstract

**Background:**

The major complication faced by patients with chronic static spinal cord injury (SCI) is the loss of mobilization. With the aim to rehabilitate SCI patients, physiotherapy is performed worldwide. However, it only helps the patients to live with their disabilities. An interdisciplinary management involving human embryonic stem cell (hESC) therapy along with physiotherapy as a supportive therapy offers regenerative treatment of the patients with SCI.

**Main body:**

The present study focuses on the role of physiotherapy in the mobilization of patients with SCI (paraplegic 136; tetraplegics 90) undergoing hESC therapy. At admission, patients were assessed on the basis of clinical and American Spinal Injury Association Impairment Scale (AIS), where 153, 32, 36 and 5 patients were designated with AIS score A, B, C and D, respectively. After 8–12 weeks of hESC therapy and physiotherapy, the patients showed clinical and scoring improvement. The patients with AIS score A shifted to B (15.0 %) and C (37.3 %), whereas, patients with grade B moved to C (40.6 %) and D (3.1 %). Patients with AIS score C and D shifted to grade D (13.9 %) and E (60.0 %), respectively. Moreover, orthotic devices were reduced to simpler ones.

**Conclusion:**

The physiotherapy aided in training of cells and took care of atrophy of limbs, whereas hESC therapy resulted in an overall improvement of the patients with SCI.

**Electronic supplementary material:**

The online version of this article (doi:10.1186/s40169-016-0122-5) contains supplementary material, which is available to authorized users.

## Background

Spinal cord injury (SCI) is defined as the incidence of temporary or permanent sensory and/or motor deficiency due to occurrence of an acute abrasion of the neural elements present in the spinal canal, i.e., spinal cord and cauda equine [1]. Depending upon the severity and location of injury on the spinal cord, the patients confront partial or complete loss of sensory, autonomic and motor functions below the level of injury. The incomplete and complete loss of sensory and/or motor functions injury found below the neurological level, including the lowest sacral segment, is referred to as an incomplete and complete injury, respectively [2].

Every year, SCI degrades the quality of life of around 40–80 people per million population around the world [3, 4]. Among adults, male-to-female ratio of at least 2:1 is reported. Mortality risk is about 2–5 times higher in people with SCI as compared to the unaffected people [2]. As per the report on the causes of SCI from the National Spinal Cord Injury Statistical Center, vehicle accidents (39.08 %) are the most common cause of SCI followed by falls (29.54 %), acts of violence (14.41 %), sports and recreational activities (8.39 %) and other causes (8.57 %) that include medical surgical complications, pedestrian, hit by falling/flying object [5].

**Fig. 1:**
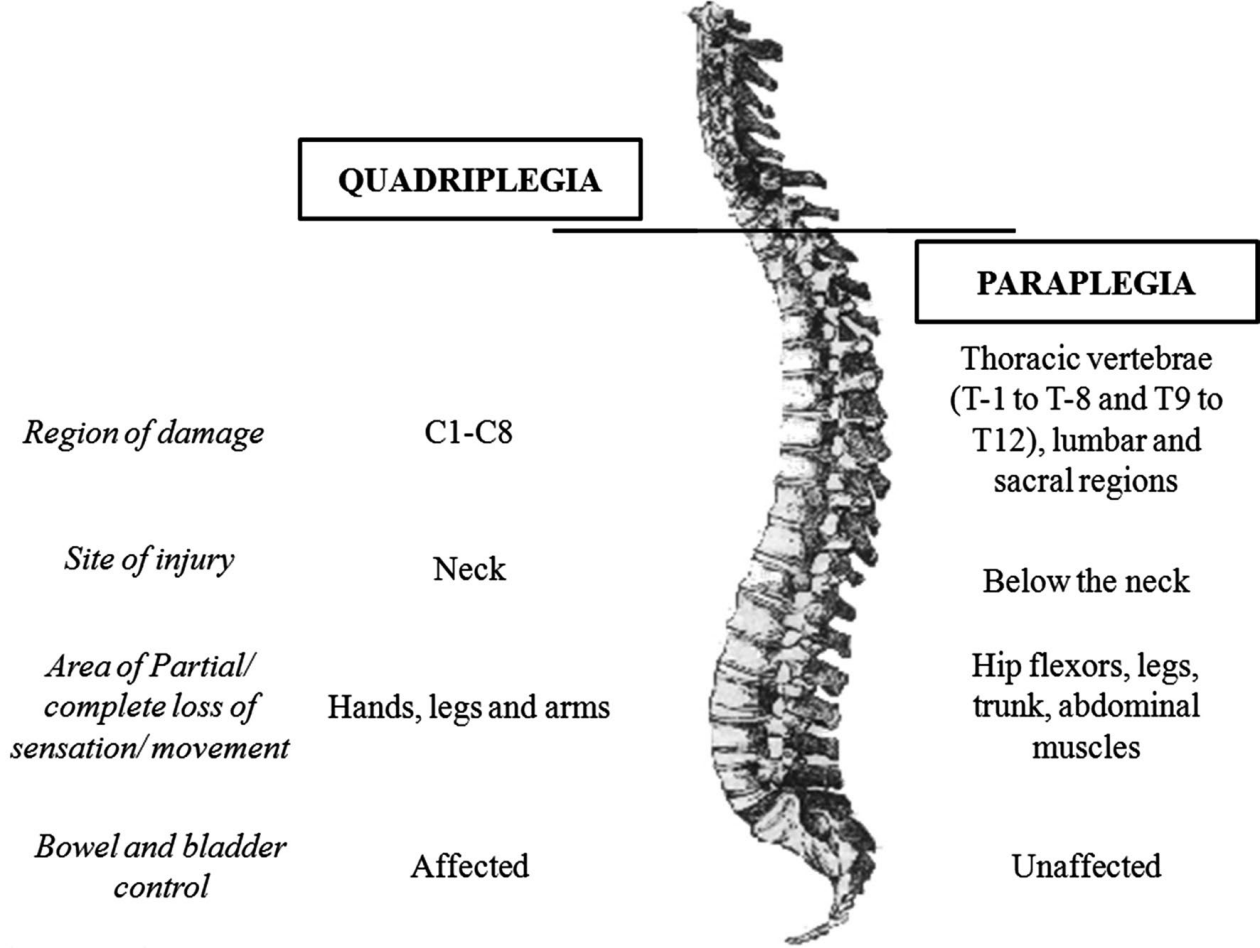
Tetraplegia and paraplegia

Based on the level of injury, patients with SCI are referred as tetraplegics (quadriplegics) or paraplegics. Tetraplegia refers to a damage to the cervical spinal cord segments at levels C1–C8, whereas, injury to brachial plexus lesions is not included. Paraplegia includes injury to cauda equina and conus medullaris, thoracic vertebrae (T-1 to T-8 and T9 to T12), lumbar and sacral regions of the spinal column, but excludes injury to the lumbosacral plexus lesions (Fig. 1) [6]. Injury to the peripheral nerves outside the neural canal is not included in any of them [7]. It has been reported that since 2005, about 40.85, 21.6, 21.4 and 15.8 % of the patients with neurological disorders were affected with incomplete tetraplegia, complete paraplegia, incomplete paraplegia and complete tetraplegia, respectively [1].

Management therapies attributed to tetraplegia and paraplegia involve the use of steroids, pain medications, surgery, bed rest, etc. [7, 8]. Stem cell therapy using neural stem cells (NSCs), adult stem cells such as mesenchymal stem cells (MSCs), etc. has been investigated as a promising tool for the treatment of SCI [9]. Human embryonic stem cells (hESCs) have a huge potential of self renewal, pluripotency and unlimited propagation [1, 10, 11]. In previous studies, the beneficial effects of hESC therapy have been reported in patients with chronic SCI [12].

Physiotherapy helps individuals with SCI to be able to function with their injuries in a day to day situation. It involves exercising for mobilization as well as for stimulation of the nerves and muscles below the level of the injury [13, 14]. Besides, it may also help in restoring the functioning of atrophied muscles [15, 16]. Though largely helpful, it only helps the patients with SCI to live with their injury and to prevent further deterioration. The interdisciplinary management of SCI involves hESC treatment as well as physical therapy (as supportive therapy) for rehabilitation of the patients with SCI. The twin-benefit, i.e. the retraining of the muscles during physiotherapy as well as the regeneration of the spinal cord due to hESC, is the likely reason that the patients have shown benefits in their clinical condition [12, 17], the present study focuses on the role of physiotherapy in the mobilization of patients with SCI receiving hESC therapy.

## Cases

The cases diagnosed with chronic static SCI elsewhere were admitted at Nutech Mediworld. On assessment with American Spinal Injury Association Impairment Scale (AIS), they were classified as paraplegic or tetraplegic. Prior to admission, they underwent intensive rehabilitative physiotherapy at their local centers, which had not led to any improvement in their functional capacity. A total of 226 patients (mean age 28 years; age range 20–34 years; male:female = 167:59), out of which 136 were paraplegic, whereas, 90 were tetraplegic, were given the physiotherapy along with hESC therapy (primary therapy). Their medical history revealed that all the patients were chronic patients with SCI for more than 1 year. They received hESC therapy as described in previous papers along with the physiotherapy which involved various exercises and use of mobility aids, depending upon their site and level of injury [12]. The patients were handled by skilled and experienced physiotherapists.

## Framework to formulate a physiotherapy program

The physiotherapy program primarily focused not only on the rehabilitation of the patients, but also to improve their quality of life. The treatment phase varied in cases with paraplegia and tetraplegia from 8 (average days: 62) to 12 weeks (average days: 73), respectively. The physiotherapists followed some key steps to formulate the process of physiotherapy to achieve highest rate of success with minimized failures.

### Assessment of complication and its level

The medical history or presence of any deformity in patients was recorded along with the determination of vital parameters such as blood pressure, pulse rate, temperature, weight, height, etc. The assessment of superficial and deep sensation also took place by defining the area of pain. The reflexes at plantar, ankle, abdomen, biceps, triceps and knee were also accessed. The presence/absence as well the extent of co-ordination problem were also analyzed. The ability to balance while sitting and standing without/with the help of calipers was also reviewed. Additional file 1: Appendix presents the manual which was followed to examine the muscles, plantar reflex, type of caliper required, the need of mobility aid, bladder and bowel sensation/control, etc. The entire assessments were video-graphed.

On the basis of AIS scores, 67.70 % (n = 153) patients were designated with AIS score A, whereas, 14.16 % (n = 32) patients were assessed with AIS score B. The number of patients with SCI who scored AIS grade C and D were 36 (15.93 %) and 5 (2.21 %), respectively [18].

### Goal planning

A comprehensive and effective physiotherapy program is required for the complete rehabilitation of the patient which involves goal planning. The goals were articulated by the patient as per their requirement and were finalized by the multidisciplinary team including clinicians and physiotherapists [14].

### Physiotherapy

The patients undergoing hESC therapy started taking physical therapy as a support therapy. It included various exercises, gait training as well the use of devices for positioning of joints and the mobility aids.

#### Exercises

 Exercise is important for patients with SCI. A wide range of exercises were practiced by paraplegics and tetraplegics depending upon the level of injury.

*(i) Range of motion (ROM) exercises*: Patients with SCI at T10 and above are usually ambulated for exercise [19]. ROM exercises are of two types, i.e., active and passive. Active (self) ROM exercises included the exercises performed by the patient himself such as stretching; the patient uses his strong arm that helps the weaker arm to perform exercises. Studies report the use of stretching as an effective way to treat and prevent contractures [20, 21]. Passive ROM exercises needed the assistance of a physiotherapist. They were performed within the ROM to avoid fatigue, swelling and pain. Though, they did not strengthen the muscles, but, helped in avoiding stiffness of joints and maintained functional capacity [22].

ROM exercises helped the patients in preservation as well as improvement in the flexibility and mobility of the joints. They also reduced the stiffness and risk of freezing of joints during the progression of disease.

*(ii) Isometric, active or active-assisted truncal exercises*: The patients with partial movement in joints/incomplete injury were made to perform these exercises for early mobilization. Recent studies have also shown that early mobilization plays an important role in the prevention of pulmonary function decline and aid in muscle strengthening [23, 24].

*(iii) Empowering exercises*: Empowering exercises are the active and resistance exercises which were performed by patients with SCI to restore the strength of upper extremities and shoulder rotation required for swimming, using electric bicycles and walking [25].

*(iv) Weight and resistance exercises*: The muscle strength of paraplegics and tetraplegics was improved by weight and resistance exercises. These exercises were performed using dumbbells in bed, the weight of which is dependent on the muscle strength of the patient [13].

In addition, breathing exercises were also performed by complete or incomplete paraplegic and tetraplegic patients to protect their lung capacity and strengthen their upper extremities. Generally, tetraplegics suffer from impaired chest wall and diaphragm muscle function which leads to an improper functioning of the lungs, subsequently causing death. Therefore, regular airway clearance therapy was essential [23, 24, 26].

*(v) Gait training*: Gait training is provided to patients with SCI to encourage them to stand and walk where ever possible. Gait cycle is divided into two phases, i.e. stance and swing. The ratio of time consumption by each phase is 3:2, where stance and swing occurs when the foot is on and off the ground, respectively [26].

*(vi) Use of parallel bars*: People with an “incomplete” SCI, irrespective of the level of injury, have more potential to regain walking than those with a “complete” SCI. Therefore, parallel bars were used for ambulation to achieve truncal and pelvic stabilization in the patients. Once the patients were able to stand upright, either with/without a posterior shell, a blend of standing, balancing and moving exercises were performed with the support of a posterior shell in the parallel bars and a long-locked knee joint walking device to ensure the integrity and stability of the lower extremity joints in patients [27].

*(vii) Mobility aids*: The patients with SCI are usually asked to use walkers or wheelchairs for mobility. Depending upon the need and severity of injury, the wheelchairs were customized in terms of dimensions such as the height, pelvic width, seat length, backrest, seat and arm support. The customization of wheelchairs (as per patient’s requirement) allowed optimal mobility of the patients, protection of their skin integrity and maintenance of normal anatomical posture. In cases with affected upper extremities, a battery assisted wheelchair was used, whereas, a manual wheelchair was preferred for the patients with injury at lower levels. However, wheelchairs lead to secondary complications due to long term sitting [28]. A modification of wheelchair, i.e., TEK robotic mobilization device is a mobility aid which offers a significant improvement in mobilization over wheelchairs [29].

**Fig. 2:**
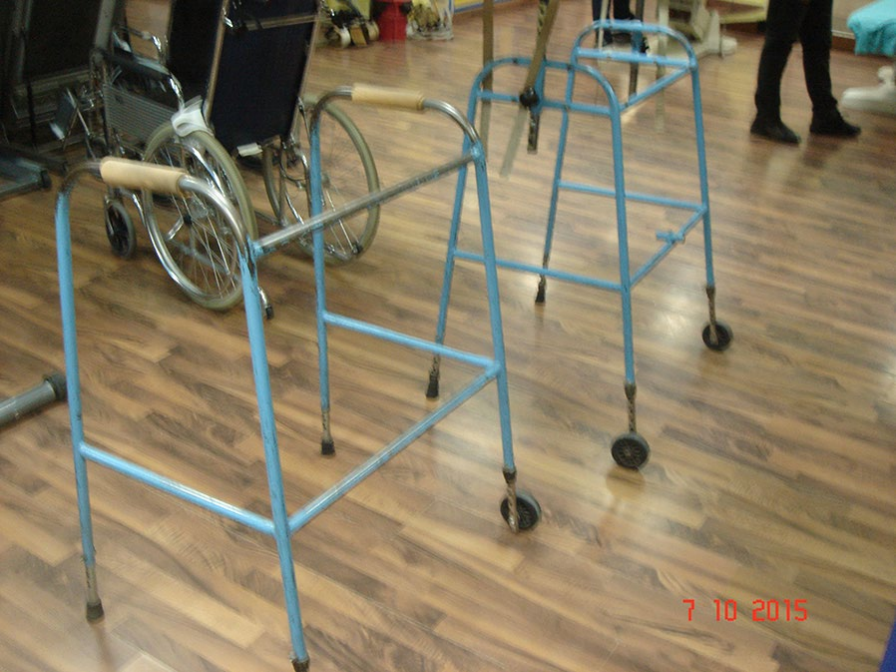
Wide walker

**Fig. 3:**
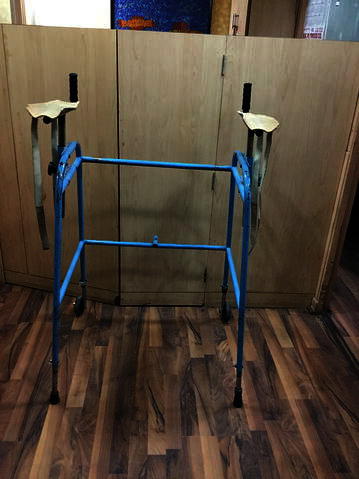
Wide walkers with an elbow support

Walkers or walking frames (Fig. 2) are considered more stable than other mobility aids because of their large base. Their center of gravity also falls within the base [26]. The walkers were also designed, keeping in mind, the level and severity of injury. Figure 3 represents a wide walker with an elbow support. Such walking frames were recommended to the tetraplegic patients. For patients who had lost hand grip, the hands could be tied with a strap to the walker. These straps could be removed on improvement of the hand grip.

**Fig. 4:**
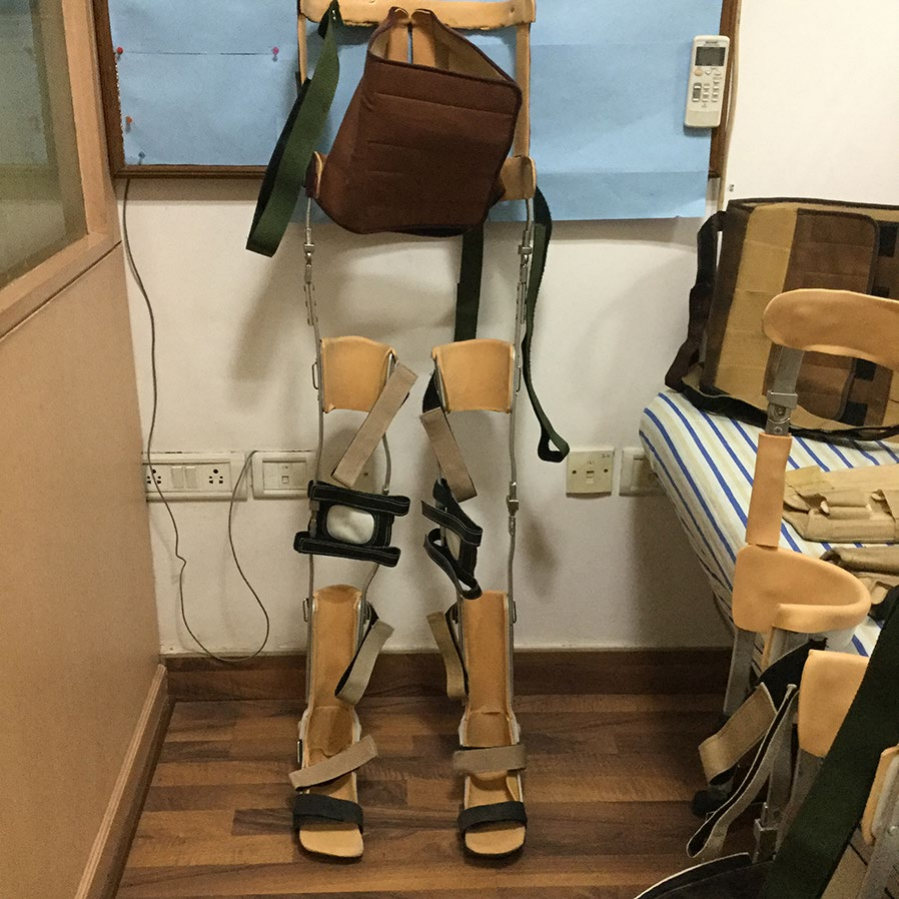
Thoracic hip knee ankle foot orthosis (THKAFO)

**Fig. 5:**
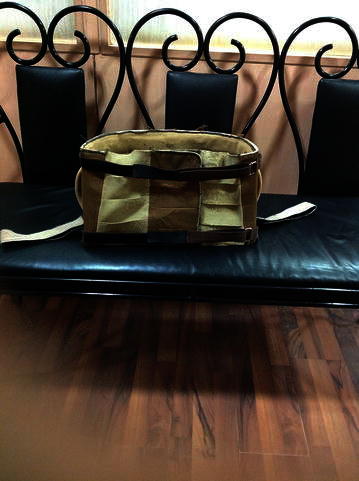
Hip knee ankle foot orthosis (HKAFO)

**Fig. 6:**
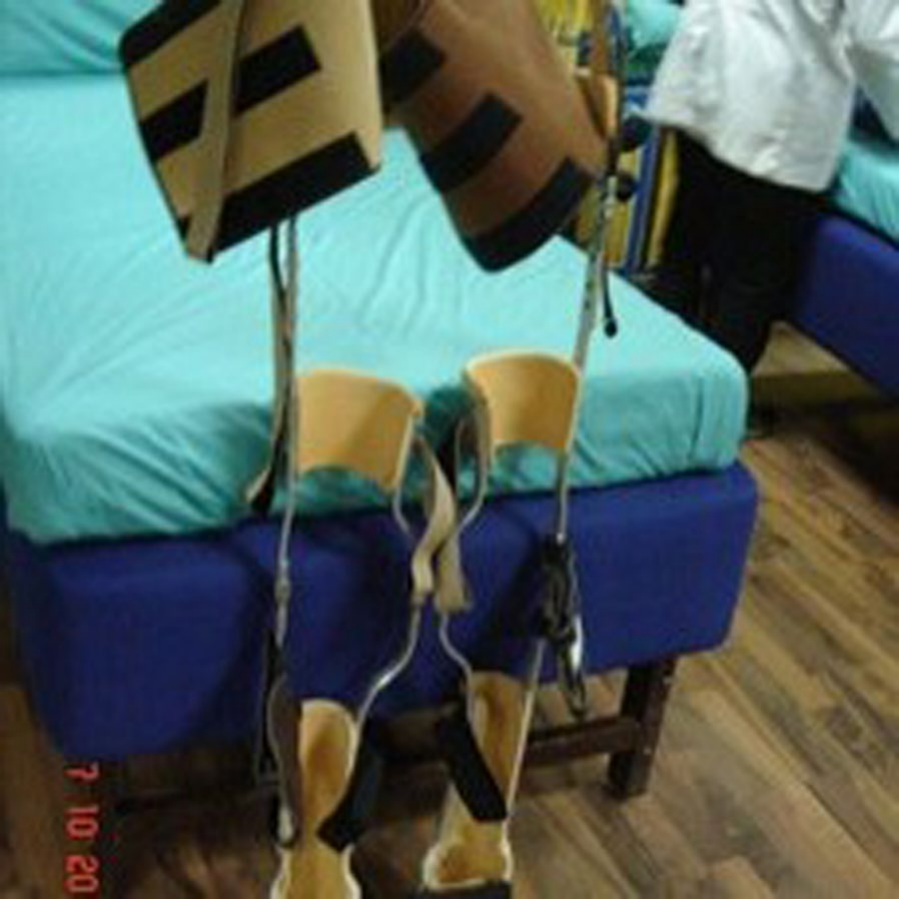
Shannon’s brace

*(viii) Positioning of the joints*: Positioning of the joints plays an important role in the mobilization of the patients with SCI. In order to protect the articulary structure and maintain the optimal muscle tonus; sand bags, pillows, plaster splints or more rigid orthotic devices were used [30]. The patients were prescribed with orthosis, depending upon the severity of injury, the extent of deformities, contractures and stiffness. An improvement in the patients (after hESC therapy and physiotherapy) led to the modification/reduction in the orthotic devices. The tetraplegics were recommended with trunk–hip–knee–ankle–foot orthosis (THKAFO). THKAFOs (Fig. 4) are prepared by attaching a thoracic extension to knee–ankle–foot orthosis (KAFO). They help the patients in controlling the hip region as well as in supporting the trunk and the lower limbs [31]. As the hESC treatment progressed, an improvement was observed in the patients with tetraplegia which led to a change in the type of caliper. The caliper was reduced to hip–knee–ankle–foot orthosis (HKAFO; Fig. 5). The further improvement, in terms of stability and balancing, observed in patients reduced the caliper to a KAFO with a Shannon’s brace. Shannon’s brace is a customized waist belt tied onto the thoraco-lumbar region. It has been named after the first patient who used it (Fig. 6). Strengthening of the thoraco–lumbar region was the major advantage associated with the use of Shannon’s brace as it did not provide support to the pelvis portion of the patient. Subsequently, the patients were able to walk without brace. Further improvement in the patients led to a reduction to knee extension brace with an ankle–foot orthosis (AFO) followed by Enrique brace. Enrique brace is a gentler/simpler knee brace which is also named after the patient who used it for the first time. This knee brace can rotate at the level of knees and gives more flexibility to the patients.

**Fig. 7:**
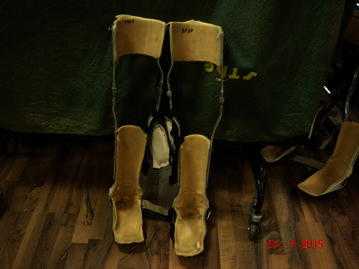
Knee-ankle–foot orthosis (KAFO)

**Fig. 8:**
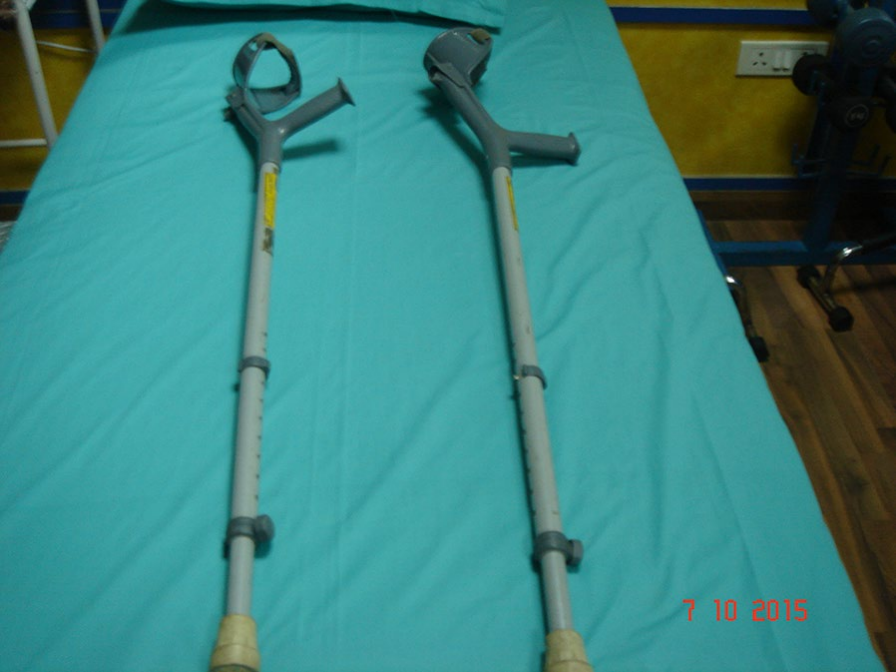
Elbow crutch

The patients with paraplegia were made to stand with full support on a HKAFO. The orthotic devices were modified and designed depending upon the condition and requirement of the patients. The paraplegics were assessed on similar lines as tetraplegics. As connectivity was regained, the support was reduced to KAFO (Fig. 7) followed by a knee brace and an ankle support. Subsequently, upon improvement, the patients just used ankle support for standing and walking. The walking aids also reduced from walkers along with manual support to just a walker. The patients showed improvement which reduced the orthotic devices to crutches (Fig. 8). Further, only walking sticks were used and finally no aid was needed.

Tetraplegics as well as paraplegics initiated their journey of standing and walking with the help of a walker. They were further reduced to wide walkers. Subsequently, they were replaced by normal walkers. When the patients improved further, they were able to walk with the help of a stick.

After every 4–6 months, (at each level), the patients were individually examined to assess the level of improvement, on the basis of which calipers were reduced. They were designed and customized depending upon the level of deformity and pathological condition of the patient. Different materials were used for the preparation of the orthotics which included aluminium, aluminium alloy, thermosetting plastic, iron, leather, rubber, foam, velcro, silicon, etc.

### Measurement of outcomes and success

The physiotherapists, with the help of the clinicians, measured the level of improvement in the patients with SCI. A patient’s status, in terms of mobilization and improvement, was videographed on weekly basis to assess the progress in posture, balancing, standing, sitting, walking, etc. The patients were assessed with AIS impairment scale for the assessment of improvement in patients. Besides, the tractographic images via magnetic resonance imaging (MRI) also showed improvement in the patients with SCI.

## Discussion

SCI is an injury of the spinal cord which ranges from foramen magnum to the cauda equine [32]. In response to the “primary damage” caused to the patient, some defense mechanisms, such as hemorrhage, inflammation and release of various chemicals, take place which lead to “secondary damage” [33]. The severity of the SCI depends upon the extent as well as the level of injury resulting in barriers to self dependence in terms of physical, functional, psychosocial and economic status.

The site of injury and region affected after SCI might cause tetraplegia or paraplegia. Tetraplegia refers to impaired functioning of the arms, trunk, legs and pelvic organs, whereas, paraplegia affects the trunk, legs and pelvic organs [34]. Besides this, many complications originate due to the hindrance in the normal activity such as improper bladder and bowel movement, urinary tract infections, pressure ulcers, orthostatic hypotension, fractures, deep vein thrombosis, spasticity, autonomic dysreflexia, pulmonary and cardiovascular problems, and depressive disorders. However, immobilization of the joints is one of the most common complications faced by patients with SCI [35, 36]. The major cause of immobilization is the spasticity which occurs in about 70 % of patients with SCI. It is also associated with the muscle paralysis, reduced muscle tone and absent tendon reflexes below the level of injury [37, 38].

Though, the treatment/management via pharmacological interventions plays a major role in the rehabilitation of patients with SCI, a support through non-pharmacological measures is also important. An interdisciplinary approach is applied for the rehabilitation of patients with SCI, which includes a team consisting of clinicians skilled in hESC therapy and physiotherapists.

The AIS score based outcomes of these patients have been previously presented [18]. Overall, the patients (n = 153) with AIS score A showed an improvement to score B (n = 23; 15 %) and C (n = 57; 37.3 %). However, a total of 73 (47.7 %) patients did not show improvement as per AIS scoring system, but a clinical improvement was observed in them. In the patients (n = 32), who scored AIS score B at the time of admission, were improved to AIS score C (n = 13; 40.6 %) and D (n = 1; 3.1 %). There was no change in the grade of 18 (56.3 %) patients, though a clinical improvement was observed in all of them. The patients with AIS score C (n = 36) and D (n = 5) showed an improvement to AIS score D and E in 5 and 3 patients, respectively.

The present study focused on the role of physiotherapy in improving the mobilization in patients with SCI receiving hESC therapy. It reveals an improvement in mobilization in patients with chronic SCI after receiving a combination of hESC and physical therapy. The physical therapy aided in training of cells and took care of atrophy of limbs, whereas hESC therapy resulted in an overall improvement of the patients. This has been observed due to the reduction in the orthotic devices and use of mobility aids. A previous study also showed remarkable improvement in the clinical, locomotive as well as functional symptoms of the patients, where, 81.72 % were able to walk with the support of calipers and mobility aids after receiving hESC therapy [12].

**Fig. 9:**
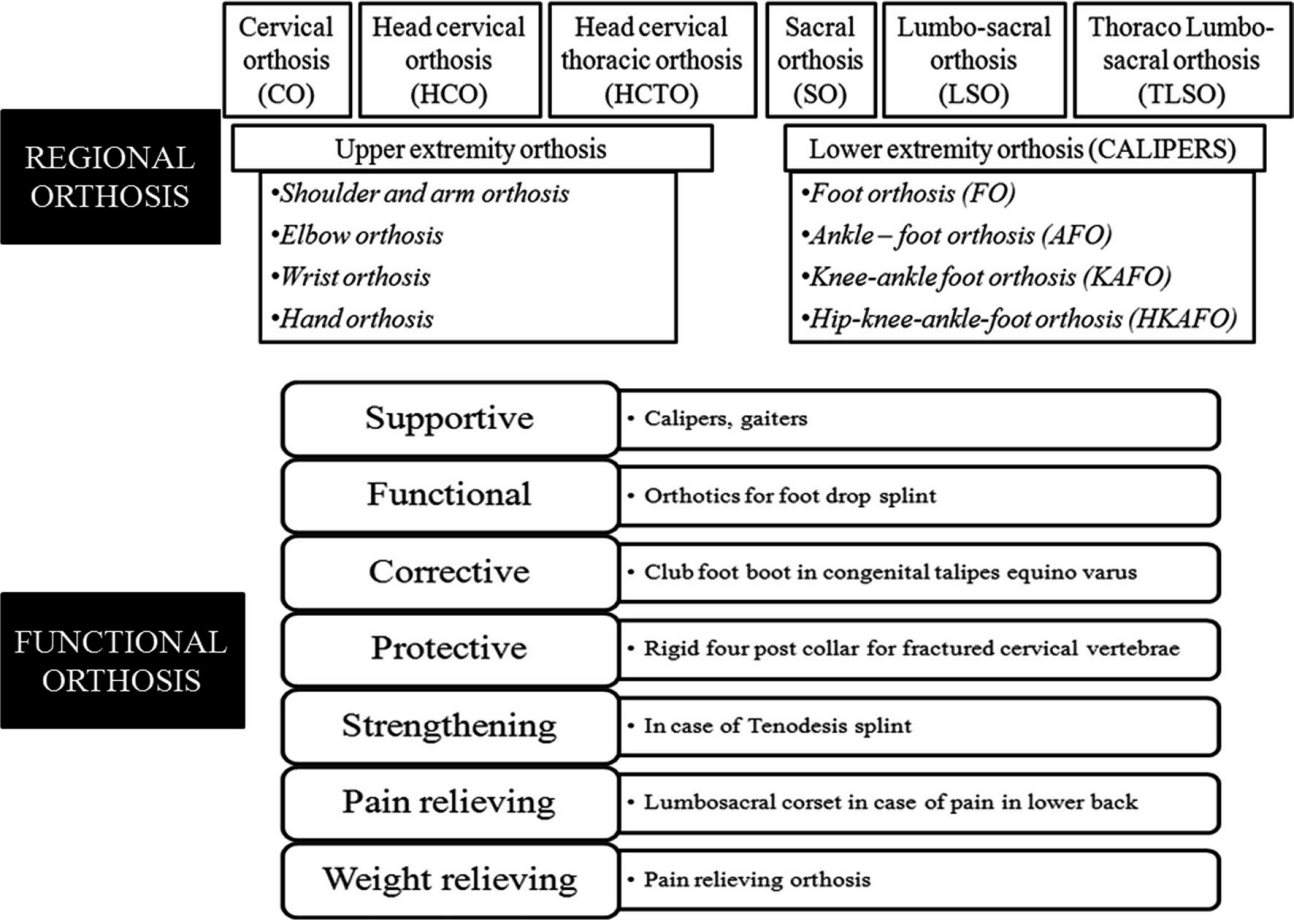
Classification of regional and functional orthosis

Orthosis are mechanical, externally applied devices which are fit to the body parts for the restoration and maintenance of their anatomical and functional position [26]. According to International Standards Organization (ISO), they are used to modify the structural and functional characteristics of the neuromuscular and skeletal system [28]. The functional and regional classification of orthosis has been explained in Fig. 9.

Designing of a well fit orthotic device is important as it serves to be beneficial to patients with SCI undergoing chronic stage ambulation, thereby enhancing the therapeutic effect. Therefore, customized orthotic devices differ from the standard devices and are customized as per the need of the patient. The aim of physiotherapy should focus to make the patient independent as far as possible. To achieve this, strengthening of the weaker muscles rather than just supporting them should be the major goal. Moreover, an individual assessment should be performed as per patient’s condition and deformity to customize the mobilization therapy.

The devices used for some of the patients are also customized, sometimes designed and manufactured by our centre. They are used by patients for standing upright, balancing, standing and walking. Besides mobilization, they limit/remove the risk of deformities, overstretching and contractures, as well as maintain the anatomical/functional position of the extremity. Studies also report the advantages of orthosis for primary management of SCI which involve the benefits associated with standing, walking for short distances, etc. [39].

Calipers are the orthotic devices used in SCI cases with injury at T6–T9. The classification of calipers has been mentioned in Fig. 9. They are fitted to the lower limb of SCI patients with the help of metal rods. KAFO is a type of orthotic device which stabilizes knee, ankle and foot, where the knee is supported by a caliper. In some cases, the joint is locked using a drop lock, spring loaded lock, cam lock, ball lock, dial lock or a plunger type of lock. The KAFOs are mainly used to support the patients with muscle weakness, upper motor neuron lesions and lost structural integrity [40]. However, combination of KAFO and a Shannon’s brace provides better support. Shannon’s brace is used separately or combined with KAFO to provide waist support, once the patient shows improvement. It helps the patients in restoring his functional abilities. In context to locomotion, it helps a patient to become independent of supportive/mobility aids.

The support to the weak musculature around the ankle joint is provided by AFOs. AFOs also help in gait exercise by stabilizing the joint for effective push-off during late stance, preventing toe-drag during swing. They also minimize the risk of falling as well as enhance the walking ability by providing safe joint mechanics [32, 40, 41]. The AFOs should be designed support the ankles only and should not provide support to the calf muscles.

The improvement in the balance control, sitting and standing postures observed after the treatment with hESC therapy lead to a modification/alteration in devices. The custom made orthotic devices are modified by altering their support system, shape or size. Such a modification approach might help in improving the ability of the patients with SCI rather than in modifying their environment.

## Conclusion

Reduction of pain and swelling, improvement in motion, improvement in gross and fine motor skills, and relaxation and healing of soft tissues is the main focus of physiotherapy. Still, it helps the patients to live with their disabilities. The regenerative capacity of SCI translating into improvements in quality of life in patients with chronic SCI seems to be very encouraging. The hESC therapy along with physiotherapy which addresses the regeneration that is going on in the patient could herald a new approach in the treatment of SCI.

## Additional file

**Additional file 1: Appendix.** Parameters for assessment of patients with spinal cord injury.

## Abbreviations

SCI: spinal cord injury
NSCs: neural stem cells
hESCs: human embryonic stem cells
AIS: American Spinal Injury Association Impairment Scale
ROM: range of motion
THKAFO: trunk–hip–knee–ankle–foot orthosis
KAFO: knee–ankle–foot orthosis
AFO: ankle–foot orthosis
HKAFO: hip–knee–ankle–foot orthosis
MRI: magnetic resonance imaging
ISO: International Standards Organization
