# Expression of Concern to: Role of physiotherapy in the mobilization of patients with spinal cord injury undergoing human embryonic stem cells transplantation

**DOI:** 10.1186/s40169-017-0166-1

**Published:** 2017-09-18

**Authors:** Geeta Shroff, Dipin Thakur, Varun Dhingra, Deepak Singh Baroli, Deepanshu Khatri, Rahul Dev Gautam

**Affiliations:** 1 Nutech Mediworld, H‑8, Green Park Extension, New Delhi, 110016 India; 2 Nutech Mediworld, New Delhi, 110016 India

## Expression of Concern: Clin Trans Med (2016) 5:41 DOI 10.1186/s40169-016-0122-5

The Editors-in-Chief of *Clinical and Translational Medicine* are issuing an editorial expression of concern to alert readers that concerns have been raised regarding the ethics of this study [1]. Appropriate editorial action will be taken once this has been fully investigated. Geeta Shroff disagrees with this notice. Dipin Thakur, Varun Dhingra, Deepak Singh Baroli, Deepanshu Khatri and Rahul Dev Gautam have not responded to our correspondence about this article.
