# Supplementary material for: Role of physiotherapy in the mobilization of patients with spinal cord injury undergoing human embryonic stem cells transplantation
Source: Clin Transl Med. 2016 Oct 20;5:41. doi: 10.1186/s40169-016-0122-5 (PMC5073087; doi:10.1186/s40169-016-0122-5)
Supplement: Supplementary file 1 — Additional file 1: Appendix. Parameters for assessment of patients with spinal cord injury. [file 40169_2016_122_MOESM1_ESM.docx]

**Appendix 1:** Parameters for assessment of patients with Spinal cord injury

| **GENERAL ASSESSMENT** | | | |
| --- | --- | --- | --- |
| **History of present illness** |  | | |
| **Past surgical/ medical history** |  | | |
| **Salient points seen in assessment** |  | | |
| **Video recording** | Yes  *By:*  *Date:* | No | |
| **VITALS** | | | |
| **Blood pressure** |  | | |
| **Pulse rate** |  | | |
| **SPO_2_** |  | | |
| **Temperature** |  | | |
| **Respiratory rate** |  | | |
| **Height** |  | | |
| **Weight** |  | | |
| **INITIAL ASSESSMENT** | | | |
| **Superficial sensation** (Define area) | Pain till:  Temp till:  Light touch till: | | |
| **Deep sensation present till** | Name of the area: | | |
| **Reflexes**  Plantar  Ankle  Biceps  Triceps  Knee reflex |  | | |
| **Any deformity** |  | | |
| **Co-ordination problems** |  | | |
| **Sitting balance** | Static:  Dynamic: | | |
| **Standing balance** | Static:  Dynamic: | | |
| **Was it possible to stand earlier** | Yes | | No |
| **With calipers** | Yes  *Full/ Thigh/ Knee/ AFO* | | No |
| **Bed sores present** | Yes  *Number:*  *Size:*  *Area:* | | No |
| **CLINICAL ASSESSMENT** | | | |
| **Plantar reflex** |  | | |
| Extensor |  | | |
| Extensor fanning |  | | |
| Flexor and fanning |  | | |
| Normal |  | | |
| Absent |  | | |
| **Calipers** (THKAFO/ HKAFO/ KAFO/AFO/other ) |  | | |
| **Mobility aid** |  | | |
| **Balance** |  | | |
| Sitting with/ without support |  | | |
| Standing with/ without support |  | | |
| **Type of gait** |  | | |
| **Posture** |  | | |
| **Bladder sensation (**Full/ partial) |  | | |
| **Control (**Nil/Full>5min/ Full>10min/ Partial<1min) |  | | |
| **Voiding sensation (**Nil/ Full/ Partial) |  | | |
| **Bowel sensation (**Full/ Partial) |  | | |
| **Evacuation (**Full/ Partial) |  | | |
| **Control (**Nil/ >1 min/ >5 min/ >10 min) |  | | |
| **Tremors (**Resting/ intentional) |  | | |
| **Tremors** (Whole body/ half body/ One limb/ Occasional) |  | | |
| **Speech (**Sounds/ Incomprehensible/ Slurred) |  | | |
| **Sleep (**Excessive/ Less/ Daytime drowsiness) |  | | |
| **Writing (**Cannot write/ Font size changed/ No problem) |  | | |
| **Clonus: U/L (**All the time/ Off and on/ Only on movement) |  | | |
| **Clonus: L/L (**All the time/ Off and on/ Only on movement) |  | | |
| **Breathing (**On ventilator/ On bipap/ SpO_2_ <90%) |  | | |
| **Swallowing (**On peg tube/ Liquids only/ Solids/ Semi solids) |  | | |
| **Mood (**Depressed/ Anxious/ Motivated/ Too bubbly/ Normal) |  | | |
| **Breathlessness (**<100 meters/ On climbing up stairs (2 flights)/  On sitting) |  | | |
| **Palpitation (**<100 meters/>100 meters/ Climbing up stairs/ Stationary) |  | | |
| **Drooling (**All the time/ Occasional) |  | | |
| **Abnormal sensation** |  | | |
| **Pain (Name body part)** |  | | |
| **VAS score and character** |  | | |
| **Frequency (**Full day/ Few times a day/ Occasional) |  | | |
| **Co-ordination** |  | | |
| **Non equilibrium (**Finger to nose/ Sliding heel on opposite shin) |  | | |
| **Equilibrium (**Feet together/ On one foot with eyes open/ On one foot with eyes closed/ Walk on straight line with eyes open/ Standing with one foot in front of the other) |  | | |

| **MANUAL MUSCLE TESTING** | | |
| --- | --- | --- |
| **Left** | **Muscle Group** | **Right** |
| ***Upper limb: Shoulder*** | | |
|  | Flexors |  |
|  | Extensors |  |
|  | Abductors |  |
|  | Adductors |  |
|  | External rotator |  |
|  | Internal rotator |  |
| ***Upper limb: Elbows*** | | |
|  | Flexors |  |
|  | Extensors |  |
|  | Supinators |  |
|  | Pronators |  |
| ***Upper limb: Wrist*** | | |
|  | Palmer flexors |  |
|  | Dorsi flexors |  |
|  | Radial deviators |  |
|  | Ulnar radiators |  |
| ***Upper limb: Fingers*** | | |
|  | Flexors |  |
|  | Extensors |  |
|  | Abductors |  |
|  | Adductors |  |
| ***Lower limb: Hip*** | | |
|  | Flexors |  |
|  | Extensors |  |
|  | Abductors |  |
|  | Adductors |  |
|  | External rotator |  |
|  | Internal rotator |  |
| ***Lower limb: Knee*** | | |
|  | Flexors |  |
|  | Extensors |  |
| ***Lower limb: Ankle*** | | |
|  | Dorsi flexors |  |
|  | Plantar flexors |  |
|  | Inverters |  |
|  | Evertors |  |
| ***Lower limb: Toes*** | | |
|  | Flexors |  |
|  | Extensors |  |
| ***Grip*** | | |
|  | Slight bending of fingers |  |
|  | Bending of fingers and holding |  |
|  | Bending of finger and weak grip |  |
|  | Full grip |  |
| **OTHER PARAMETERS** | | |
|  | ***Spasticity*** |  |
|  | U/L |  |
|  | L/L |  |
|  | ***Spasticity with clonus*** |  |
|  | U/L |  |
|  | L/L |  |
|  | ***Spasticity with deformity*** |  |
|  | U/L |  |
|  | L/L |  |
|  | ***Flaccidity*** |  |
|  | U/L |  |
|  | L/L |  |
|  | ***Flaccidity with muscular contracture*** |  |
|  | U/L |  |
|  | L/L |  |
|  | ***Bulk/limb atrophy*** |  |
|  | U/L |  |
|  | L/L |  |
|  | ***Sensation*** (Superficial/ deep) |  |
|  | ***Temperature*** (Hot/ cold) |  |
|  | ***Pain*** (Deep stimuli/ pin prick) |  |

^U/L: Upper limbs; L/L: Lower limbs; THKAFO: Thoracic hip knee ankle foot orthosis; HKAFO: Hip knee ankle foot orthosis; KAFO: Knee ankle foot orthosis; AFO: Ankle foot orthosis^
